# A framework for antecedents of social entrepreneurial intention: Empirical evidence and research agenda

**DOI:** 10.3389/fpsyg.2022.988851

**Published:** 2022-10-13

**Authors:** Sabine Bergner, Carolin Palmer, Megan Devaney, Philipp Kruse

**Affiliations:** ^1^Department of Psychology, University of Graz, Graz, Austria; ^2^Institute for Business Administration and Leadership (IBAL), University of Applied Sciences, Cologne, Germany; ^3^Department of Psychology, University of Limerick, Limerick, Ireland; ^4^Faculty of Psychology, Technical University Dresden, Dresden, Germany

**Keywords:** social entrepreneurship, social entrepreneur, entrepreneurial intention, antecedents, theory of planned behavior, South Africa

## Abstract

Social entrepreneurship (SE) increasingly contributes to diversity in entrepreneurship. The different approaches to SE suggest a variety of antecedents which drive individuals' intention to become social entrepreneurs. While this variety of antecedents is insightful, it also creates a need for systemisation and prioritization. We address this need by introducing an integrative, multi-level framework for person-based antecedents of SE-intention. Based on this multi-level framework the antecedents are grouped on three theoretical levels which refer to an individual's (1) personality, (2) cognition, and (3) entrepreneurial exposition. When testing our framework with 499 South African University students we find support for the multi-level framework and its notion that antecedents from the diverse levels complement each other. Therefore, this study provides a structure for person-based antecedents of SE-intention and additionally points to future research which may extend the proposed framework.

## Introduction

Social entrepreneurship (SE) is widely acknowledged as an effective tool to address the increasing discrepancy between the very top and the very bottom of societies (Wilkinson and Pickett, 2009; European Commission, 2013). It works by blending financial and social value creation (Austin et al., 2006), fosters innovation and financial independence of stakeholders (Dupuy et al., 2016) and further positively influences individuals, groups, and societies (Kickul et al., 2018; Cinar, 2019). Due to its benefits, various programmes have been launched to foster social entrepreneurship. The majority of these programmes promotes the individual *intention* to become a social entrepreneur, as this intention is considered the single most important predictor of founding a social enterprise (Hockerts, 2017; Kruse, 2020a).

There is large consensus that SE-intention is strongly driven by person-related antecedents such as values, motives, or personality traits (Sastre-Castillo et al., 2015; Bacq et al., 2016; Saebi et al., 2019). Yet, three central limitations blur this consensus. First, the number of person-based antecedents for SE-intention is enormous and thus difficult to overlook (Nga and Shamuganathan, 2010; Wachner et al., 2015; Kruse, 2020a). In fact, the large quantity of antecedents impedes navigation through the field and further bares the risk of an “inability to build cumulative knowledge” (Venkataraman, 1997; p. 135). Second, most of the studies investigating antecedents of SE-intention focus on a single theoretical model (Short et al., 2009; Sassmannshausen and Volkmann, 2018). It goes without saying that SE is an interdisciplinary phenomenon and thus various theoretical models have to be considered conjointly to understand its antecedents. Finally, there is—at least to our knowledge—no agenda that specifically guides future *empirical* research on antecedents of SE-intention, a circumstance that clearly impedes progress in the field. In essence, SE-research seems to lack (i) an integrative framework of person-based antecedents of SE-intention which is (ii) empirically supported and allows deriving (iii) a distinct research agenda for future studies in the field.

The significance of this study is threefold. First, as research matures in social entrepreneurship, greater attention to theory building regarding its antecedents becomes a priority. Theory building enhances the field and is best done when drawing on systemised and structured knowledge (Shepherd and Suddaby, 2017). Currently, a systemised and structured overview of person-based antecedents regarding SE-intention is missing, preventing advancements in theory building. This study presents a theoretically grounded systematization of person-based antecedents of SE-intention and thus helps to enable theory building in the field. More detailed, it systemises the most prominent person-based antecedents alongside the distal-proximal-motivation framework of Kanfer (1990) and assigns them to the level of personality, cognition, or entrepreneurial exposition.

Second, this study offers an empirical validation of the systematization framework according to which the antecedents are structured. Thus, it goes beyond sheer theoretical reasoning and suggests that the person-based antecedents of SE-intention are indeed grouped on different levels. More detailed, a large-scale sample of South African students shows that antecedents from different levels complement each other when predicting a person's intention to launch a social enterprise.

Finally, this study enhances theory building by identifying three particularly promising streams for future (empirical) SE-intention research which are derived on the basis of this study's empirical insights. In fact, we derive specific research questions which are thought to inspire future theory and research on SE.

## Theoretical background

### Social entrepreneurship as a new form of entrepreneurship

Social entrepreneurship (SE) is considered a new form of entrepreneurship which deliberately incorporates a social mission into a business model (Austin et al., 2006; Wry and York, 2017). The social mission can be diverse and includes but is not limited to alleviating poverty or integrating marginalized groups into the labor market (Perrini et al., 2010; Mittermaier et al., 2021). As both—the social mission and the income-oriented business model—are combined, social enterprises are also referred to as hybrid enterprises (Tracey and Phillips, 2007; Kruse et al., 2021). Importantly, while acting on a social mission is possible for any business, including for-profit and non-profit enterprises (Borzaga and Santuari, 2003), social enterprises focus on self-financing their social actions and on remaining independent from political or private donations. As a result, social enterprises are likely to be perceived as apolitical and more sustainable than for instance NGOs. This is a major advantage as political neutrality helps to avoid governmental interference (Dupuy et al., 2016) and higher sustainability supports the enterprise's independence even in times of crises like the COVID-19 pandemic when donations are commonly cut down (Branas-Garza et al., 2020).

In essence, SE can be conceptualized as a new, hybrid form of entrepreneurship which combines the fulfillment of a social mission with the aspiration to generate monetary profit and to self-finance the social actions (Kruse et al., 2021). Therefore, SE is largely seen as a hybrid form of entrepreneurship bridging the gap between for-profit-only enterprises and traditional NGOs (Lepoutre et al., 2013). The increasing interest in social enterprises builds on their great potential to contribute to a more just and equal society.

### Social entrepreneurial intention

Behavioral intentions are the single most important predictors of any planned behavior and explain about 28% of its variance (Sheeran, 2002). Importantly, this also holds true for the entrepreneurship context where a clear link between entrepreneurial intention and action was found (Kautonen et al., 2015). While entrepreneurial intention is an important prerequisite of entrepreneurial activity (Krueger and Brazeal, 1994; Kolvereid and Isaksen, 2006), *social* entrepreneurship intention determines *social* entrepreneurship activity. In this regard, social entrepreneurial intention refers to a person's determination to plan a new social business and to consciously set it up at some point in the future (Thompson et al., 2000).

Whether individuals intend to become a social entrepreneurs strongly relies on their person-based characteristics such as personality traits, cognitive skills, or individual values (McClelland, 1961; Steward, 1996). Two strategies have been applied to pin down person-based antecedents of SE-intention. First, antecedents are derived from theoretical models transferred from other disciplines to the field of SE. An example thereof is the Theory of Planned Behavior (Ajzen, 1991) which is used in but not limited to the context of entrepreneurship. Second, antecedents are drawn from theoretical models specifically developed for SE. An example thereof is the Model of Social Entrepreneurial Intention Formation by Mair and Noboa (2006) which was specifically developed for the context of social entrepreneurship. Subsequently, we summarize the most prominent person-based antecedents of SE-intention alongside the theoretical models they originate from and finally structure them according to a new integrative, multi-level framework.

### Person-based antecedents of SE-intention and the theories they originate from

#### SE-intention antecedents proposed by the theory of planned behavior

The TPB (Ajzen, 1991) is one of the most wide-spread theories to predict entrepreneurial intention and behavior (Chipeta et al., 2016; Gorgievski and Stephan, 2016). According to the theory's assumption, any planned behavior relies on the intention to perform it. In the context of SE, intention formation is thought to be influenced by (1) attitudes toward SE, (2) subjective norms regarding SE, and (3) perceived behavioral control. According to Ajzen (1991), a negative attitude toward SE describes the negative evaluation of becoming a social entrepreneur and will decrease the probability of becoming one, whereas a positive evaluation will increase this probability. Subjective norms reflect normative beliefs that signify the influence of others on personal decisions in personal life. For instance, if friends approve SE-activities then the probability of performing these activities will increase. Finally, perceived behavioral control refers to a person's self-efficacy to successfully perform entrepreneurial behavior and to its perceived controllability. Thus, perceived behavioral control is high if individuals consider themselves capable of starting and managing a social enterprise and if they see themselves capable of controlling relevant aspects. Recent empirical findings demonstrate that the antecedents postulated by the TPB are valid in the SE-context and influence a person's intention to found a social enterprise even across different cultures and economic circumstances (Yang et al., 2015; Cavazos-Arroyo et al., 2017; Tiwari et al., 2017).

Important for this study, the TPB suggests a so-called “thinking–doing link” (Mitchell et al., 2007) and thus stresses a cognitive approach to entrepreneurship. Accordingly, in order to *do* something individuals have to *think* of their actions beforehand. Naturally, this approach highlights the individual thinking and decision-making processes (Mitchell et al., 2002) which is why all antecedents derived from the TPB are considered as *cognitive antecedents* of SE-intention.

#### SE-intention antecedents proposed by the model of social entrepreneurial intention formation

A second approach frequently applied in SE is the Model of Social Entrepreneurial Intention Formation by Mair and Noboa (2006). This model suggests that empathy, moral judgement, self-efficacy, and social support are direct antecedents of SE-intention which, in turn, triggers actions relevant to found a social enterprise. While *empathy* refers to the ability to cognitively understand and affectively share the emotional situation of others, *moral judgement* denotes the motivation to help others to create a common good. Both, empathy and moral judgement, enhance the attractiveness of careers in SE and in turn increase the intention to pursue such careers. Additionally, *self-efficacy* describes the conviction of being able to found a social enterprise while *social support* refers to the expected help of others when striving for a career in SE. Conjointly, self-efficacy and social support increase a person's conviction to successfully perform as a social entrepreneur. In line with the model's assumption, all four antecedents directly predict the intention to found a social enterprise (Bacq and Alt, 2018; Dickel and Eckardt, 2020).

Similar to the TPB, Mair and Noboa's model highlights the cognitive elements of SE-intention. Important for this study, while the TPB explains the intention formation processes in a wide variety of settings but is not limited to the social entrepreneurial one, Mair and Noboa's model is exclusively developed for *social* entrepreneurship. Therefore, it includes only those antecedents which are thought to be of relevance for nascent social entrepreneurs. Due to the cognitive nature of the antecedents proposed by Mair and Noboa, we also see them as cognitive antecedents, yet we account for their conceptual proximity to SE-intention which is why we consider them as SE-specific or so-called *second-level* cognitive antecedents.

#### SE-intention antecedents proposed by the basic human values theory

**Figure 1:**
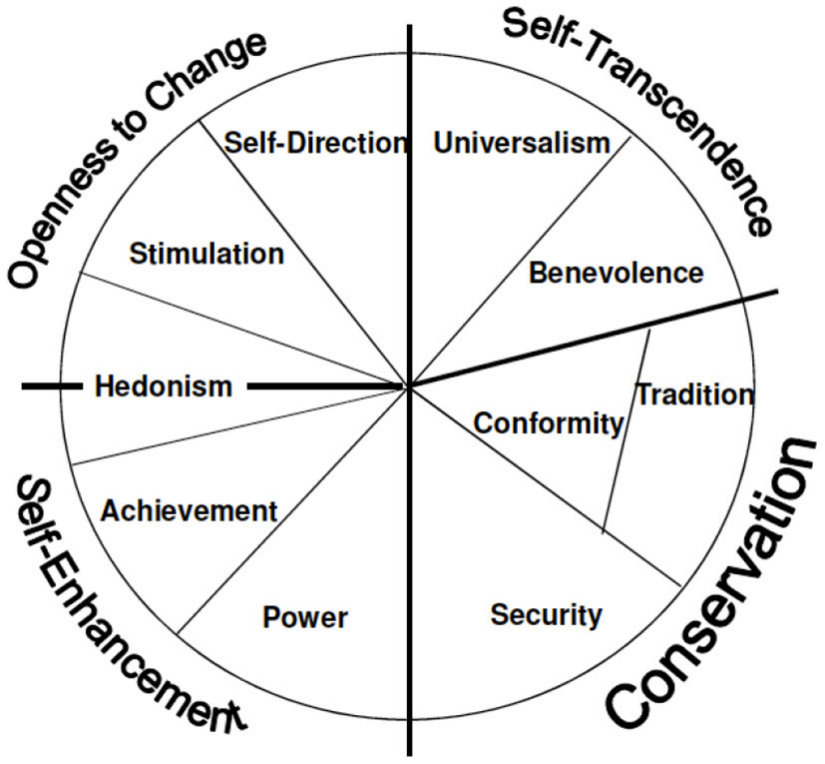
The integrated structural model of personal values (Schwartz, 2003).

The Basic Human Values Theory of Schwartz (1992, 2003) is the third theoretical approach frequently used to describe antecedents of SE-intention. Accordingly, differences in people's values are responsible for their varying professional goals and varying intention to pursue a SE career. Schwartz distinguishes ten values which are arranged in a circular model according to their similarity. More similar values are located more closely to each other and are grouped into one of the following higher-order values: (1) self-transcendence, (2) openness to change, (3) conservation, and (4) self-enhancement (Figure 1).

The higher-order value *self-transcendence* refers to benevolence and universalism and emphasizes the importance and willingness to help others. As social entrepreneurs aim at creating value to fight social challenges (Austin et al., 2006; Mair and Marti, 2006), self-transcendence values are meant to foster SE-intention. *Openness to change* refers to self-direction and stimulation as open persons enjoy free thinking, are innovative, and seek new experiences. Because this kind of self-direction and stimulation is prototypical for entrepreneurial tasks, *openness to change* is also meant to enhance SE-intention. *Conservation* describes a person's aspiration to maintain the status quo, preserve traditions, and live a secure life. This value opposes entrepreneurial tasks which commonly include risk-taking, breaking with tradition, and exploiting novel opportunities. Consequently, individuals who express high conservation values presumably express reduced SE-intention. A similar logic applies to Schwartz's last higher-order value. *Self-enhancement* refers to an individual's aspiration to reach goals which strongly match personal interests. As the focus on self-interest and personal achievement opposes the social entrepreneurs' mission to create social value, a high level of self-enhancement should impede the intention to pursue a career as social entrepreneur. In line with this reasoning, there is growing evidence that all four integrated values are valid antecedents of an individual's SE-intention (Sastre-Castillo et al., 2015; Kruse et al., 2019).

Importantly but in contrast to the previous models, Schwartz's model does not refer to cognitive antecedents of SE-intention. As values rather reflect the personality than the cognition of (nascent) social entrepreneurs we refer to them as personality-driven antecedents. Personality-driven antecedents are commonly regarded as more distal, exerting their influence on SE-intention *via* the more proximal cognitive antecedents (Kanfer, 1990), a proposition that was recently confirmed in the context of SE (Kruse et al., 2019).

Next to the three models summarized above, there are several single-constructs which are regularly discussed as important antecedents of SE-intention. These include the personality traits proactivity, risk-taking, and altruism as well as the experience-based antecedents SE-knowledge and SE-experience. These single-construct antecedents are subsequently outlined and their relation to the previously listed antecedents is discussed.

#### Single-construct antecedents of SE-intention

##### Proactive personality

Bateman and Crant (1993) define proactive personality as a “relatively stable tendency to affect environmental change” (p. 103). People who are proactive consider themselves as change agents who actively shape their environment instead of passively waiting for change to happen. Proactivity is considered an important antecedent of SE-intention, as it was repeatedly linked to persons' intention to become a traditional entrepreneur (Crant, 1996; Prabhu et al., 2012), entrepreneurial outcomes (Kickul and Gundry, 2002) and *social* entrepreneurship intention (Chipeta et al., 2016, 2022). As proactivity is a relatively stable personality trait and thus similar to Schwartz's values it will also be considered a personality-driven antecedent.

##### Risk-taking

Risk-taking is a key element in entrepreneurship and meta-analytic findings show that those who are more willing to take risks report stronger entrepreneurial intention (Rauch and Frese, 2000; Simon et al., 2000). Compared to traditional entrepreneurship, risk-taking should be even more important in SE as social entrepreneurs bare the risk of failing twice—financially and in their social mission. While traditional entrepreneurs deal with financial risks alone (Dorado, 2006; Zahra et al., 2009; McCaffrey, 2018), social entrepreneurs also have to deal with high moral standards (Johnson, 2000; Wasilczuk and Łuński, 2014) which bare an enormous risk to backfire even when only slightly bent for the benefit of financial goals (Palmer et al., 2019). Consequently, SE is closely tied to risk-taking for why individuals with higher willingness to take risks should also be more drawn to careers in SE and should thus express higher SE-intention. Given that risk-taking is largely considered as a personality trait, we take the view that risk-taking is on the same conceptual level as proactivity and Schwartz's personality-driven values. Thus, it is an antecedent on the personality level.

##### Altruism

Altruism is the tendency to generously and kindly help others without or with low-scale external incentives (Rushton et al., 1981). Altruistic reasoning was spotted as a strong motivational driver for SE (Mair and Marti, 2006) and is further regarded as one of the most important traits of social entrepreneurs (Tan et al., 2005). Similar to risk-taking, proactivity and Schwartz's values, altruism is considered a relatively stable and rather general personality trait. Consequently, it also represents the personality level of antecedents.

##### SE-knowledge and SE-experience

Knowledge about and experience with certain careers prevent from unrealistic career expectations (Gati et al., 1996) and facilitate career decisions (Lease, 2004). This holds also true for the field of SE and turns the business experience as well as experience with social problems into relevant antecedents of SE-intention (Hockerts, 2017; Bacq and Alt, 2018). Consequently, knowledge about and experience with SE will be considered as important drivers for SE-intention in this study. However, compared to the previously mentioned cognitive and personality-related antecedents, SE-knowledge and experience are highly specific for SE and provide the most detailed information on a future career as a social entrepreneur. Therefore, we take the view that knowledge and experience are very proximal antecedents of SE-intention with a larger effect on SE-intention than the previously presented antecedents on the personality and cognitive level.

### A multi-level framework for systemising antecedents of SE-intention

As shown, entrepreneurship research provides a rich diversity of antecedents for SE-intention. Although this diversity is fruitful for the development of SE theory (Osiri et al., 2019), it also impedes navigation through the field which bares the risk of an inability to create cumulative knowledge for theory building (Venkataraman, 1997). While this risk was generally spotted in entrepreneurial research—independent of whether the focus was set on social or general entrepreneurship—effort to address it was primarily put into *general* entrepreneurship (see Gorgievski and Stephan (2016), Zhao et al. (2010), and Alferaih (2017) for notable examples). In contrast and according to recent research (Weerakoon, in press), the effort for systemising antecedents of *social* entrepreneurship falls comparably short.

The main reason why antecedents of SE-intention still lack systematization might lie in the fundamental disparities between general and social entrepreneurship which directly affect the motivational drivers thereof (see Austin et al. (2006) for an overview). Keeping in mind that social and general entrepreneurship differ and that their person-based intentional drivers differ, makes a sheer adaption of findings from general to social entrepreneurship inappropriate. Findings by Wach et al. (in press) strengthen this argument and show substantial differences in person-based antecedents of the intention to launch a general vs. social enterprise. These differences are particularly clear when it comes to personal attitudes or perceived behavioral control and appear to be globally present as they were found in different cultures. Consequently, we build on this research demonstrating that the person-based antecedents for general vs. social entrepreneurial intention differ and argue that it is thus necessary to offer a systematization framework particularly derived for antecedents of SE-intention. In fact, a structured framework allows for more detailed insights on whether antecedents are unique or redundant, complement each other or trigger each other in a processual manner. To structure the drivers of SE-intention we apply the distal-proximal-motivation framework of Kanfer (1990). This particular framework was used because it validly groups motivational antecedents (Diefendorff and Chandler, 2011), is meaningful in the setting of SE, and helps following a recent call for more systematization of antecedents in SE (Saebi et al., 2019).

**Figure 2:**
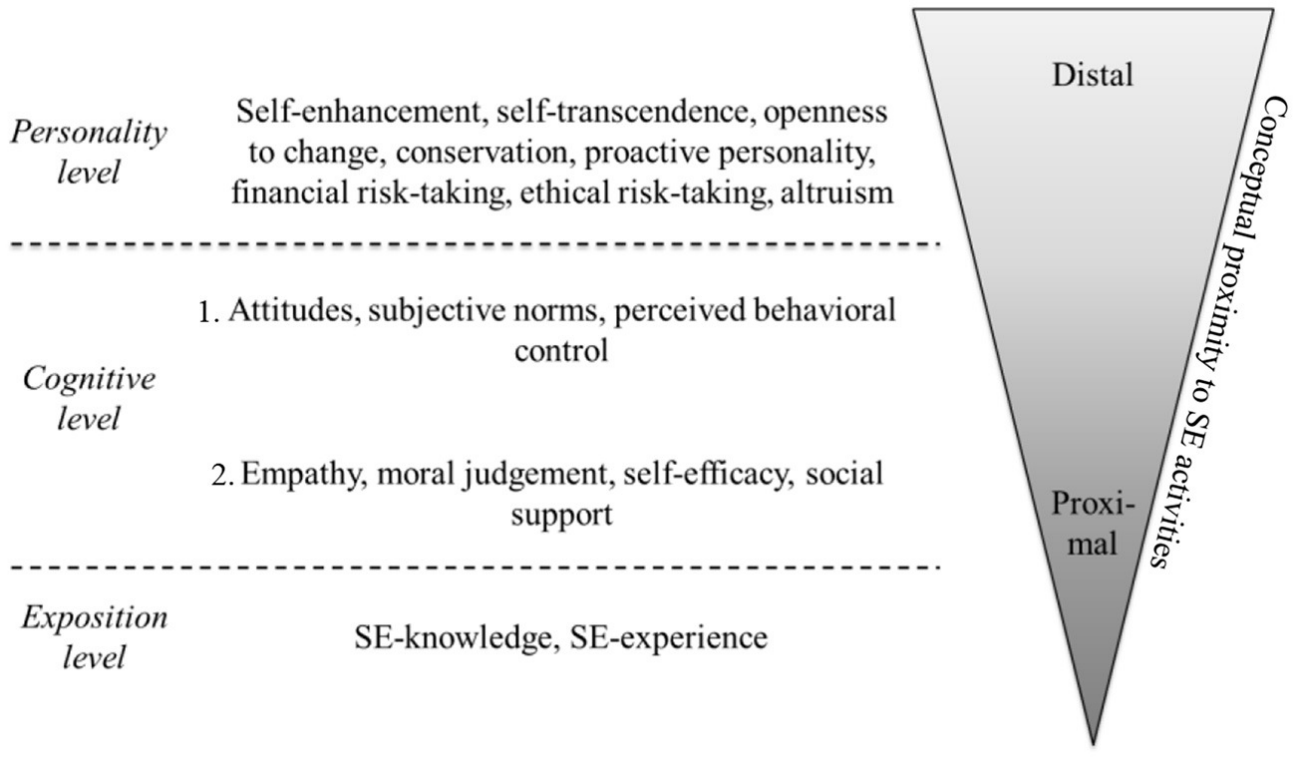
Arrangement of hypothesized Antecedents of SE-intention based on their conceptual Proximity to SE Activities. As the constructs empathy, moral judgement, self-efficacy, and social support are elements of the SE-specific model by Mair and Noboa (2006), they are perceived to be more proximal than the TPB-components attitudes subjective norms and perceived behavioral control which are applicable to a wide range of different behaviors.

In line with Kanfer (1990), we suggest that SE-intention is influenced by antecedences which can be organized according to their *conceptual proximity* to entrepreneurial actions. Proximal antecedents are narrowly defined and SE-specific. They shape a person's wish to pursue a career in SE and help to set the stage for actions in SE. In contrast, distal antecedents are more broadly defined and rather unspecific which is why they are important for a wide variety of settings including but not limited to SE. Distal antecedents exert their impact often rather indirectly through more proximal ones which is why their direct link is commonly weaker (Judge et al., 2009). Important for this study, both, more proximal and distal antecedents predict a person's intentional level *separately*. However, considering proximal and distal antecedents *conjointly* should result in the most accurate prediction of a person's SE-intention. Figure 2 depicts the antecedents of SE-intention grouped according to the distal-proximal-motivation framework.

The most distal level integrates all of Schwartz's personal values as well as the personality traits proactivity, risk-taking, and altruism. We will refer to it as the *personality level*. According to Bergner (2020), the personality of an individual reflects “the enduring set of traits and styles that he or she exhibits” (p. 4). Consequently, the common core of antecedents on the personality level is that they are relatively stable across time and situations and are usually not bound to a certain career context. They are rather distal and shape a person's career intention in diverse settings including but not limited to SE. For example, a person scoring high on the value self-transcendence will probably favor a job with social and caring tasks. However, this could result in the intention to become a social entrepreneur but also in the wish to work as a caregiver, social worker or teacher. Thus, the antecedents on the personality level drive career intentions in a rather broad and general way and compared to antecedents on the cognitive level, which are presented next, they (i) do not involve a *mental* or *intellectual* reflection of career options and (ii) are less prone to change as a result of one's own thinking process, for instance when acquiring more information about alternative career tracks (see Hueso et al. (2020) for an overview in the entrepreneurship context). In brief, antecedents on the personality level are understood as enduring, innate socio-emotional characteristics of a person.

The next level of our integrated, multi-level framework refers to antecedents of SE-intention on the *cognitive level* and comprise the components of the TPB and the model proposed by Mair and Noboa. Antecedents of this level denote a person's cognitive effort to evaluate the attractiveness of a career as social entrepreneur. As this evaluation involves critically questioning the specific tasks of social entrepreneurs and challenging one's own capabilities to successfully complete them, there is a certain proximity to the SE-intention formation process. In fact, initial empirical findings provided by Kruse et al. (2019) suggest that cognitive antecedents of SE-intention are more proximal than antecedents on the personality level. Important for this study, we see a difference between the antecedents derived from the TPB and the model by Mair and Noboa which is why we distinguish a first *and second cognitive level*. The antecedents of the TPB are applicable to a wide range of planned behaviors including but not limited to entrepreneurship and are thus more distal to SE-actions. Therefore, they are considered as antecedents on the more distal, first cognitive level. In contrast, the model of Mair and Noboa comprises solely SE-specific antecedents with a high proximity to SE-actions. Therefore, they are considered as antecedents on the more proximal, second cognitive level.

The final set of antecedents for SE-intention refers to the amount of SE-knowledge and SE-experience. It is termed the *exposition level*. The antecedents on this level are all directly linked to the targeted intention and include the active gain of SE-relevant knowledge and experience. Importantly, this knowledge and experience goes beyond the sheer cognitive assessment of an SE-career, which is reflected on the cognitive level. Consequently, the exposition level is the most proximal one.

Based on Kanfer's (1990) distal-proximal-motivation framework we propose that more proximal antecedents are not only more strongly tied to SE-intention, but also enhance the prediction of more distal ones. Translating this assumption to an empirical level means that more proximal antecedents should add incremental validity over more distal ones when predicting the intention to become a social entrepreneur. Thus, the following hypotheses (H) are stated:

*H*_1_: Antecedents on the personality level (self-enhancement, self-transcendence, openness, conservation, proactivity, risk-taking, altruism) significantly predict SE-intention.

*H*_2_: Antecedents on the cognitive level 1 (attitude toward SE, subjective norms, perceived behavioral control) incrementally predict SE-intention beyond the antecedents of the personality level.

*H*_3_: Antecedents on the cognitive level 2 (empathy, moral judgement, self-efficacy, social support) incrementally predict SE-intention beyond the antecedents of the personality and cognitive level 1.

*H*_4_: Antecedents on the exposition level (SE-knowledge and SE-experience) incrementally predict SE-intention beyond the antecedents of the personality, cognitive 1, and cognitive 2 level.

## Methods

### Data acquisition and sample

In total, 499 participants (55% female) with a mean age of 22 years (*SD* = 2.58) provided data in this study. Overall, the participants were between 17 and 35 years old (*M* = 21.53, *SD* = 2.58), 74% reported having a Black/African background whereas 10% had an Indian, 10% a White/European, 5% a Colored, and 1% a Chinese ethnical background. The majority of the participants were undergraduates (91%). Participation was voluntary, anonymous, and not incentivised.

The data was collected using a paper-pencil questionnaire which was distributed in undergraduate courses of a university in Johannesburg. Notably, the South African sample is a clear benefit for research on SE. First, it allows examining the SE-intention in a country with one of the highest SE-activity rates. Second, it represents a non-western entrepreneurial mind-set and thus increases the generalizability of findings on SE-intention which mainly build on western samples (Steckler and McLeroy, 2008; Campbell and Stanley, 2015).

### Measures

#### SE-intention as the criterion of interest

SE-intention is defined as the aspiration to found a social enterprise in one's professional career. It was measured using the Social Entrepreneurial Intention Scale of Kruse et al. (2018) where participants rate six items on a 7-point-Likert scale ranging from 1 (“not at all”) to 7 (“very much”). The following item is a sample: “I have the intention to found an enterprise that combines a social mission and an elaborated income strategy”. In this study the scale's internal consistency was 90.

#### Person-based antecedents of SE intention

##### Antecedents on the personality level

Antecedents on the personality level include the Schwartz values self-enhancement, self-transcendence, openness, and conservation and the single-construct antecedents proactivity, risk-taking, and altruism.

The Schwartz values *self-enhancement, self-transcendence, openness, and conservation* were measured using the Portrait Value Questionnaire that subsumes 19 statements which represent other people's goals in life (PVQ; Schwartz, 2003). Participants have to rate the extent to which these goals fit their own ones by using a 6-point Likert scale ranging from 1 (“not like me at all”) to 6 (“very much like me”). Four items represent the s*elf-enhancement* subscale which denotes the aspiration to achieve challenging goals and gain power in one's life (α = 0.64; sample item: “Being very successful is important to him. He likes to impress other people”). The subscale *self-transcendence* was measured by five items and refers to the aspiration to help other people and to be benevolent (α = 0.68; sample item: “She thinks it is important that every person in the world is treated equally. She wants justice for everybody, even for people she doesn't know”). The subscale *openness to change* refers to the aspiration to think freely and to be innovative and was measured with four items (α = 0.62; sample item: “It is important to him to make his own decisions about what he does. He likes to be free and not depend on others”). Finally, the five-item subscale *conservation* denotes the aspiration to keep the status quo, preserve law and order, and live a secure life (α = 0.62; example item: “She believes that people should do what they're told. She thinks people should follow rules at all times, even when no-one is watching”).

*Proactive personality* refers to the disposition to act as a change agent and affect one's environment. It was measured using the Proactive Personality Scale by Bateman and Crant (1993) which consists of five items (α = 0.83). Participants rate their level of agreement on a 7-point-Likert scale ranging from 1 (“strongly disagree”) to 7 (“strongly agree”). The following is a sample item: “I can spot a good opportunity long before others can”.

*Risk-taking* was measured with the subscales (i) financial and (ii) ethical risk-taking of the Domain-Specific Risk-Taking Scale (DOSPERT; Blais and Weber (2006). Participants indicated the probability to perform certain actions on a 7-point-Likert scale ranging from 1 (“extremely unlikely”) to 7 (“extremely likely”). *Financial risk-taking* defines the willingness to invest money in a risky manner and was assessed with three items (α = 0.74; sample item: “Investing 10% of your annual income in a new business venture”). *Ethical risk-taking* describes the willingness to perform actions widely considered as immoral and was measured with six items (α = 0.72; sample item: “Not returning a wallet you found that contains $200”).

*Altruism* denotes the disposition to help others despite no or just minimal external incentives. It was measured using the Altruism Scale by Rushton et al. (1981) which asks study participants to indicate the frequency of six behavioral items (α = 0.70). Answers were provided on a 5-point-frequency scale ranging from 1 (“never”) to 5 (“very often”). The following is a sample: “I have donated goods or clothes to a charity”.

##### Antecedents on the first cognitive level

Antecedents on the first cognitive level include all components of the TPB attitudes. *Attitudes toward social entrepreneurship*, s*ubjective norms, and perceived behavioral control* were assessed using the Entrepreneurial Intention Questionnaire (EIQ) by Liñán and Chen (2009) in its adapted version for social entrepreneurship (Kruse, 2020a). Participants had to rate their agreement to various statements using a 7-point-Likert scale from 1 (“strongly disagree”) to 7 (“trongly agree”). *Attitudes toward social entrepreneurship* reflect the attitudes toward social entrepreneurship and are measured with five items (α = 0.88) such as “Being a social entrepreneur implies more advantages than disadvantages to me”. *Subjective norms* refer to the social pressure of trusted ones when it comes to the personal goal of becoming a social entrepreneur. It was measured with four items (α = 0.82) similar to this example item: “If I decided to create a social enterprise, my close family would approve of that decision”. Finally, *perceived behavioral control* describes the extent to which a person believes to perform as and control the process of becoming a social entrepreneur. It was assessed with six items (α = 0.90) similar to the following: “To start a social enterprise and keep it working would be easy for me”.

##### Antecedents on the second cognitive level

Antecedents on the second cognitive level include all components of Mair and Noboa's (2006) Model of Social Entrepreneurial Intention Formation. *Empathy, moral judgement*, s*elf-efficacy*, and s*ocial support* were measured using the Social Entrepreneurial Antecedents Scale by Hockerts (2015). Participants provided their agreement regarding various statements using a 5-point-Likert scale varying from 1 (“strongly disagree”) to 5 (“strongly agree”). *Empathy* describes the ability to cognitively understand others and emotionally share their feelings. It was assessed by six items (α = 0.77) similar to the following: “When thinking about socially disadvantaged people, I try to put myself in their shoes”. *Moral judgement* refers to the motivation to help others achieving a common goal and was measured with four items (α = 0.81; sample: “It is an ethical responsibility to help people less fortunate than ourselves”). *Self-efficacy* denotes the conviction that one is able to found and successfully run a social enterprise. It was measured with four items (α = 0.68) similar to the following: “I am convinced that I personally can make a contribution to address societal challenges if I put my mind to it”. Finally, *social support* describes the degree to which a person thinks that others support his/her aspiration to act as a social entrepreneur. It was assessed with four items (α =0.65; sample: “People would support me if I wanted to start an organization to help socially marginalized people”).

##### Antecedents on the Exposition Level

Antecedents on the exposition level include knowledge and experience in the context of social entrepreneurship. *SE-knowledge* describes the extent to which a person is familiar with the concept of social entrepreneurship as a career option. It was assessed with three items (α = 0.77) particularly developed for this study which are rated on a 7-point-Likert scale ranging from 1 (“not at all”) to 7 (“very much”). The following is a sample item: “I had been familiar with the term “social entrepreneur” before participating in this study”. *SE-experience* refers to the degree to which a person has already gained practical insights in the field of SE. It was measured with five items (α = 0.88) particularly developed for this study which had to be rated on the same Likert-scale. An example item is “I have already gained practical experience in the field of social entrepreneurship (e.g., during an internship)”.

#### Control variables

Sociodemographic variables impact the intention to found a social enterprise. For instance, women express higher SE-intention compared to men (Chipeta et al., 2020). Also, age and education level have been shown to affect the SE-intention formation process (Wachner et al., 2015). Consequently, we included sex, age, and educational level as controls. Furthermore, due to the ethnic diversity in South Africa (Rivera-Santos et al., 2015), the participants' ethnicity was also included as a control variable.

### Analysis strategy

To test our hypotheses, we conducted hierarchical regressions on the participants' intention to become a social entrepreneur using the software IBM SPSS 25. The control variables (age, sex, educational level, ethnicity) were entered in the first model. Subsequently, we arranged the person-based antecedents according to our multi-level framework of Figure 2 and added the antecedents on the personality level (model 2), the antecedents of the first cognitive level (model 3), the antecedents of the second cognitive level (model 4), and finally the antecedents of the exposition level (model 5).

## Results

### Preliminary analyses

Before testing the hypotheses, requirements for the hierarchical regressions and common method bias were checked. With respect to the statistical requirements, we visually inspected the histograms of all variables which confirmed the normality of the data and supported the use of a hierarchical regression analysis (West et al., 1995). Common method bias was investigated using a single factor test (Fuller et al., 2016). Studying all items conjointly in a factor analysis and limiting the number of extracted factors to just one, resulted in 16.45% of explained variance. As this level of explained variance is well below the suggested threshold of 50% (Podsakoff and Organ, 1986), there was no need to account for the common method bias in our analyses. Finally, we checked whether multicollinearity was an issue in our sample (Farrar and Glauber, 1967). The Variance Inflation Factors (VIF) ranged from 1.19 for risk-taking to 2.18 for self-enhancement and were all below the threshold of VIF = 4.00 (O'Brien, 2007). Thus, it was assumed that multicollinearity does not systematically bias the subsequent analyses.

### Descriptive analysis and bivariate correlations

The descriptive results and bivariate correlations of all study variables are displayed in Table 1. The intention to become a social entrepreneur most strongly relates to self-efficacy (*r* = 0.38, *p* < 0.01), attitude toward SE (*r* = 0.37, *p* < 0.01), self-transcendence (*r* = 0.35, *p* < 0.01), and moral judgement (*r* = 0.34, *p* < 0.01). Importantly, SE-intention relates to the respective antecedents only in a positive manner. Concerning the inter-correlations of the antecedents, small to medium-sized values were found.

**Table 1:** Means, standard deviations, and inter-correlations of all constructs included in the study (*N* = 499).

**Scale**	**Mean (SD)**	**2**	**3**	**4**	**5**	**6**	**7**	**8**	**9**	**10**	**11**	**12**	**13**	**14**	**15**	**16**	**17**	**18**	**19**	**20**	**21**	**22**
1. Age	21.00 (2.58)	−0.07	0.33^**^	−0.16^**^	0.13^**^	0.01	0.15^**^	−0.02	0.05	0.06	0.01	−0.15^**^	−0.04	0.00	−0.11^**^	0.08	0.07	0.00	0.02	0.15^**^	0.06	0.03
2. Sex	1.55 (0.05)		0.04	−0.05	−0.01	0.00	−0.07	0.23^**^	0.13^**^	0.07	0.03	07	0.17^**^	0.04	0.14	0.03	−0.18^**^	−0.24^**^	0.17^**^	−0.03	−0.06	0.13^**^
3. EDU	1.13 (0.49)			−0.01	0.13^**^	0.09	0.16^**^	0.03	0.07	0.07	0.09^*^	−0.11^*^	0.01	−0.01	−0.03	0.03	0.00	0.03	0.06	0.09^*^	0.02	0.06
4. ETH	1.53 (1.02)				−0.04	0.01	0.01	−0.05	−0.06	−0.03	0.06	0.08	−0.02	−0.06	−0.02	−0.11^*^	−0.09	0.03	0.06	0.04	0.02	−0.03
5. ATT	4.44 (1.40)					0.45^**^	0.56^**^	0.23^**^	0.31^**^	0.29^**^	0.23^**^	0.05	0.30^**^	0.22^**^	0.17^**^	0.31^**^	0.21^**^	−0.02	0.19^**^	0.28^**^	0.35^**^	0.37^**^
6. SN	5.19 (1.22)						0.31^**^	0.17^**^	0.24^**^	0.25^**^	0.33^**^	0.02	0.25^**^	0.22^**^	0.08	0.27^**^	0.09^*^	−0.05	0.17^**^	0.11^*^	0.17^**^	0.18^**^
7. PBC	3.50 (1.33)							0.03	0.19^**^	0.23^**^	0.24^**^	0.13^**^	0.18^**^	0.29^**^	0.14^**^	0.40^**^	0.22^**^	0.08	0.24^**^	0.44^**^	0.50^**^	0.27^**^
8. EP	4.08 (0.72)								0.47^**^	0.49^**^	0.25^**^	−0.02	0.47^**^	0.24^**^	0.18^**^	0.15^**^	0.14^**^	−0.32^**^	0.26^**^	0.03	−0.03	0.31^**^
9. MJ	3.93 (0.79)									0.30^**^	0.26^**^	0.06	0.40^**^	0.16^**^	0.31^**^	0.24^**^	0.13^**^	−0.15^**^	0.27^**^	0.11^*^	0.10^*^	0.34^**^
10. SE	3.98 (0.67)										0.35^**^	0.05	0.42^**^	0.33^**^	0.17^**^	0.35^**^	0.23^**^	−0.18^**^	0.21^**^	0.14^**^	0.03	0.38^**^
11. SS	3.50 (0.66)											0.10^*^	0.25^**^	0.17^**^	0.15^**^	0.29^**^	0.15^**^	−0.07	0.25^**^	0.18^**^	0.12^**^	0.20^**^
12. SEH	4.28 (1.00)												0.20^**^	0.34^**^	0.36^**^	0.25^**^	0.11^*^	0.17^**^	0.07	0.09^*^	0.05	0.04
13. ST	4.98 (0.77)													0.49^**^	0.49^**^	0.29^**^	0.15^**^	−0.28^**^	0.27^**^	0.07	0.02	0.35^**^
14. OP	4.69 (0.88)														0.25^**^	0.49^**^	0.22^**^	−0.02	0.19^**^	0.26^**^	0.18^**^	0.25^**^
15. CO	4.37 (0.86)															0.20^**^	0.02	−0.14^**^	0.15^**^	0.03	0.08	0.25^**^
16. PP	5.10 (1.06)																0.28^**^	−0.04	0.27^**^	0.32^**^	0.29^**^	0.28^**^
17. FRT	4.60 (1.37)																	0.13^**^	0.14^**^	0.17^**^	0.12^**^	0.14^**^
18. ERT	2.33 (1.08)																		−0.13^**^	0.03	0.10^*^	−0.08
19. ALT	3.47 (0.70)																			0.28^**^	0.22^**^	0.21^**^
20. SEK	3.20 (1.60)																				0.61^**^	0.12^**^
21. SEE	2.48 (1.45)																					0.12^**^
22. SEI	5.30 (1.21)																					−

Sex (1= Male; 2 = Female); *EDU*, Educational level (1 = Undergraduates, 2 = Honors, 3 = Masters, 4 = Others); *ETH*, Ethnicity (1 = Black/African; 2 = Other); *ATT*, Attitude toward SE; *SN*, Subjective Norms; *PBC*, Perceived behavioral control; *EP*, Empathy; *MJ*, Moral Judgement; *SE*, Self-Efficacy; *SS*, Social Support; *SHE*, Self-Enhancement; *ST*, Self-Transcendence; *OP*, Openness; *CO*, Conservation; *PP*, Proactive Personality; *FRT*, Financial Risk-Taking; *ERT*, Ethical Risk Taking; *ALT*, Altruism; *SEK*, SE-Knowledge; *SEE*, SE-Experience; *SEI*, SE-intention; *SD*, Standard deviation.

* *p* < 0.05;

** *p* < 0.01.

### Hierarchical regression analysis

Table 2 shows the results of the hierarchical regression analyses. To examine the hypotheses, the incremental value between the separate regression steps is considered. In the first step of the hierarchical regression, the control variables were entered. In a second step, the most distal antecedents—those on the personality level—were added. We found a significant change in *R*^2^ after including the antecedents of the personality level (model 2: Δ*R*^2^ = 0.17, *p* < 0.01) when predicting a person's SE-intention. Thus, *H*_1_ is confirmed.

**Table 2:** Summary of the hierarchical regression analysis (*N* = 499).

**Level**	**Constructs**	**Model 1 (β)**	**Model 2 (β)**	**Model 3 (β)**	**Model 4 (β)**	**Model 5 (β)**
Control variables	Age	0.01	0.01	−0.02	−0.02	−0.02
	Sex	0.13^**^	0.09	0.09^*^	0.08	0.08
	Education	0.05	0.04	0.02	0.01	0.01
	Ethnicity	−0.02	0.01	0.01	0.01	0.01
Personality level	Self–Enhancement		−0.13^*^	−0.11^*^	−0.09	−0.09
	Self–Transcendence		0.21^**^	0.16^**^	0.07	0.06
	Openness		0.04	0.05	0.04	0.05
	Conservation		0.13^**^	0.11^*^	0.10^*^	0.10^*^
	Proactive Personality		0.16^**^	0.11^*^	0.06	0.07
	Financial Risk–Taking		0.07	0.04	0.01	0.01
	Ethical Risk–Taking		0.05	0.03	0.06	0.05
	Altruism		0.07	0.05	0.02	0.03
Cognitive level I	Attitude toward SE			0.24^**^	0.20^**^	0.20^**^
	Subjective Norms			−0.04	−0.06	−0.06
	PBC			0.05	0.05	0.06
Cognitive level II	Empathy				0.05	0.05
	Moral Judgement				0.12^*^	0.12^*^
	Self-Efficacy				0.19^**^	0.19^**^
	Social Support				0.00	0.00
Expo. level						
	SE-Knowledge					−0.04
	SE–Experience					0.00
	**Δ** ***R** ^**2**^*	0.01*	0.17**	0.05**	0.05**	**0.00**

*PBC*, Perceived Behavioral Control; SE, Social Entrepreneurship; Δ*R*^2^, Difference in the amount of variance explained compared to the previous model (corrected *R*${}_{Total}^{2}$ =0.26).

All displayed β-values are standardized coefficients.

* *p* < 0.05;

** *p* < 0.01.

In a next step, the antecedents of the first cognitive level were added which led to another significant increase in the amount of explained variance (model 3: Δ*R*^2^ = 0.05, *p* < 0.01). Therefore, *H*_2_ is also confirmed, and the prediction of a person's SE-intention is improved by adding antecedents of the first cognitive level to those of the personality level. Subsequently, including the more proximal antecedents of the second cognitive level resulted in a further increase of explained variance (model 4: Δ*R*^2^ = 0.05, *p* < 0.01) and offered support for *H*_3_.

Finally, adding the antecedents of the most proximal level in model 5, the exposition level (SE-knowledge and SE-experience), did not result in an increase of explained variance. Thus, the prediction of a person's SE-intention cannot be further improved by adding antecedents of the exposition level to those of the previous levels. Therefore, *H*_4_ was not supported. When all antecedents were considered conjointly, 26% of the variance in a person's SE-intention was explained.

## Discussion

This study provides three main results. First, it demonstrates that the manifold person-based antecedents of SE-intention can be structured using a multi-level framework which differentiates them according to their conceptual proximity to entrepreneurial intentions. Applying this framework offers a way to systemise and integrate the rather fragmented research body of antecedents of SE-intention. Second, this study initially validates the multi-level framework by empirically supporting its underlying assumptions in a country known for its lively SE community. Third, this study's findings clearly suggest an agenda for future research when it comes to antecedents of SE-intention which is subsequently outlined.

### Assessment of a multi-level framework for antecedents of SE-intention

Our findings reveal that Kanfer's (1990) distal-proximal-motivation logic is an eligible basis to structure the most frequently discussed person-based antecedents of SE-intention. In fact, all frequently studied antecedents could be integrated. Importantly and anew, our findings suggest that the person-based antecedents represent four quite diverse categories which differ regarding their proximity to SE-intention. Moreover, our findings offer an initial explanation for why some antecedents are more strongly linked to SE-intention than others as it seems to be the relative proximity to SE-intention that affects the empirical link between the antecedents and SE-intention.

To validate the multi-level structure of our newly proposed framework, we used hierarchical regressions and analyzed a large South African sample. Our hypotheses 1–3 suggested that (i) simultaneously considering antecedents of different proximity levels provides better prediction of SE-intention and (ii) that antecedents of the personality, first and second cognitive level each bear information about a person's intention to become a social entrepreneur which is not provided by antecedents of the other proximity levels. Including the antecedents of the personality level, first cognitive level, and second cognitive level repeatedly resulted in an increase of explained variance in SE-intention and confirmed hypotheses 1–3.

Regarding our empirical findings, hypothesis 4 was not supported and antecedents on the exposition level did not enhance the prediction of SE-intention. One reason therefore might be found in research on general entrepreneurship, where previous entrepreneurial exposure is also regarded as an important facilitator for entrepreneurial intention. However, the effect of work experience in a small or newly founded firm on entrepreneurial intention is mediated by positive attitudes toward entrepreneurial careers and perceived behavioral control (Zapkau et al., 2015). Correlations from Table 1 indicate a similar pattern for SE-intention. Though knowledge of and experience with SE (exposition level) are the most proximal antecedents linked to SE-intention, their contribution to predicting SE-intention might be encapsulated in mediating variables on cognitive level 1. In addition, SE is still a relatively new phenomenon and more specific measures are needed to assess both SE-knowledge and SE-experience before final conclusions on their relative importance can be derived, especially cultural effects are expected (Zapkau et al., 2017).

### Fields for further research in SE-intention—A research agenda

In light of our newly proposed multi-level framework for antecedents of SE-intention, we take the view that it may serve as a solid scientific underpinning for future research in the field. The following research streams seem particularly promising:

#### A. Identification and investigation of SE-intention formation mechanisms

In line with our framework, we found that adding cognitive antecedents to personality-driven ones leads to a more accurate prediction of SE-intention. The distinct mechanisms underlying this finding have only rarely been investigated. However, first evidence suggests that both the personality-driven and cognitive antecedents separately and directly affect SE-intention and, even more interestingly, personality-driven antecedents affect SE-intention *via* cognitive ones (Kruse et al., 2019; Chipeta et al., 2022). Given that our multi-level framework identifies more than just personality-driven and cognitive levels, it becomes obvious that the relation between diverse antecedents is still ill understood. Nevertheless, in line with our results it can be assumed that there are overlaps as well as interdependencies among the various antecedents on the same level and across different levels. Consequently, we consider a thorough investigation of the following questions as essential to further understand the complex interplay between SE-intention antecedents:

1. How do the various antecedents on the same conceptual level relate to each other? What does their internal structure look like? Is the cognitive antecedent “Attitudes Toward Se” an independent predictor of se-intention or is it rather a mediator which triggers other cognitive antecedents on the same level?
2. Do distal antecedents on the personality level indirectly affect SE-intention *via* more proximal antecedents on the first and second cognitive level?

Furthermore, we consider our framework an open framework that allows to add a wide variety of constructs on each level, for instance, the Big Five Personality Traits (Nga and Shamuganathan, 2010). Thus, we explicitly encourage scholars to contribute to the empirically validated extension of our multi-level framework. Ultimately, this will help to get a thorough understanding of the SE-intention formation process.

#### B. Cultural embeddedness of SE-intention antecedents

The vast majority of samples investigating SE-intention stems from so called *WEIRD*-countries which are *w*estern, *e*ducated, *i*ndustrial, *r*ich, and *d*emocratic (Henrich et al., 2010). This holds true even though the biggest need for and activity in SE is found in developing countries (Ebrashi and Darrag, 2017; Najafizada and Cohen, 2017). Based on the circumstance that cultural differences between *WEIRD* and developing countries exist and that they affect SE-intention (Kedmenec and Strašek, 2017), we encourage scholars to pay more attention to a country's culture when studying SE-intention. Consequently, the following question should be addressed:

3. Can our proposed multi-level framework with its innate assumptions be applied in different cultures, i.e., Is it cross-culturally solid?

Even though our investigation is a first step toward a more culturally diverse investigation of the antecedents for SE-intention as it uses an African sample, more work is needed to gain a better understanding of the cultural dependence across antecedents and their impact. To gain such understanding we suggest conducting studies with samples from multiple countries and cultures (Gupta et al., 2020; Kruse, 2021).

#### C. Contextualizing individual-level processes in SE-intention formation

In addition to our person-based perspective on SE-intention antecedents, Institutional Theory suggests that also contextual circumstances like economy and society influence (entrepreneurial) decision making processes (Scott, 1995; Kibler et al., 2014; Kruse et al., 2021). Regarding economy, Amit and Muller (1995) distinguish between so called push entrepreneurs—entrepreneurs rather forced into their career due to a lack of alternatives—and pull entrepreneurs—entrepreneurs attracted by entrepreneurship due to its benefits. Considering the innate motivational differences comparing these two types of entrepreneurial action, the following question emerges:

4. Are different antecedents of se-intention differently important in various economic situations? For instance, are antecedents on the personality level more relevant for internally motivated pull entrepreneurs while antecedents on the cognitive level are more important for externally driven push entrepreneurs?

Regarding society, it is commonly acknowledged that the context individuals grows up in impacts the attractiveness of entrepreneurial careers (Zellweger et al., 2011; Palmer et al., 2021). Considering recent findings by Brunel et al. (2017) and Kruse (2020b) who examined the effect of role models on entrepreneurial intention, a particularly complex interplay between personality, cognitive, and social antecedents of SE-intention was found. However, the effect of social influences like a parental (social) entrepreneurship background has hardly been studied so far. Thus, the following question should be addressed:

5. To which extent does the social context (e.g., parents and role models) influence the person-based antecedents of se-intention proposed in our framework?

In addition to culture, economic drivers, and social background, the intention to found a social enterprise is impacted by gender and biological sex (Chipeta et al., 2020). Therefore, research within our multi-level framework of SE-intention antecedents should address the following question:

6. Does gender or sex impact the interaction between antecedents of different levels, for instance through gender self-concepts?

### Limitations

As with any study there are limitations to consider. First, applying a convenience sampling technique, our results are neither representative for South Africa nor for other developing countries. Furthermore, despite controlling for ethnicity in our analyses we did not explicitly account for the wide variety of different ethnicities in South Africa and their individual cultural characteristics (Rivera-Santos et al., 2015). Thus, future studies should consider individual measures of culture such as the scale proposed by Yoo et al. (2011) that assesses Hofstede's (1984) cultural dimensions on an individual level.

Second, despite the VIF not exceeding the threshold of 4.00 indicating that no notable multicollinearity problems emerged in our analysis, recent findings by Vatcheva et al. (2016) suggest that even medium-size inter-predictor correlations about 30 can cause multicollinearity-related biases undetected by the VIF. Thus, we limited our analyses to the proposed levels only and did not take the single variable effects into account.

Third, future studies might want to extend the research scope to SE-behaviors and thus offer a more comprehensive investigation on the question what affects the *actual creation* of a social enterprise. In that regard, longitudinal studies are certainly needed to close the intention-behavior-gap (Kautonen et al., 2015) and to directly link antecedents of SE-intention to observable SE-behavior (Meoli et al., 2020).

## Conclusion

This paper proposes a new multi-level framework to structure person-based antecedents of the intention to become a social entrepreneur. Based on the relative proximity to SE-intention, we identified four levels on which antecedents can be anchored: One personality level, two cognitive levels, and one exposition level. While the personality level refers to socio-emotional traits relevant for a wider variety of jobs including but not limited to entrepreneurial contexts, the cognitive level entails a person's effort to evaluate the entrepreneurial process and the exposition level includes SE-specific knowledge and experience. Empirical examination of the multi-level framework using a large South African sample provides initial support for its basic assumptions and shows that the antecedents from different levels largely complement each other. Importantly, we consider our framework an open framework that enables an empirically validated extension by adding a variety of constructs at each level, which ultimately should enable a thorough understanding of the SE-intention formation process. Finally, our findings suggest three central streams of future research which seem particularly fruitful to disentangle the twisted net of SE-intention antecedents: (1) the identification and investigation of SE-intention formation mechanisms, (2) the cultural embeddedness of antecedents, and (3) the contextualization of individual-level processes in SE-intention formation.

## Data availability statement

The raw data supporting the conclusions of this article will be made available by the authors, without undue reservation.

## Ethics statement

The current study involved human participants and was reviewed and approved at Technical University of Dresden, Germany. The participants provided their written informed consent to participate in this study. Participation was voluntary.

## Author contributions

SB, PK, and CP contributed to the conceptual idea, data analyses and write up. MD contributed to the data management. All authors contributed to the article and approved the submitted version.

## Conflict of interest

The authors declare that the research was conducted in the absence of any commercial or financial relationships that could be construed as a potential conflict of interest.

## Publisher's note

All claims expressed in this article are solely those of the authors and do not necessarily represent those of their affiliated organizations, or those of the publisher, the editors and the reviewers. Any product that may be evaluated in this article, or claim that may be made by its manufacturer, is not guaranteed or endorsed by the publisher.
